# A *PRRX1* Signature Identifies TIM-3 and VISTA as Potential Immune Checkpoint Targets in a Subgroup of Microsatellite Stable Colorectal Cancer Liver Metastases

**DOI:** 10.1158/2767-9764.CRC-22-0295

**Published:** 2023-02-09

**Authors:** Vigdis Nygaard, Anne Hansen Ree, Vegar Johansen Dagenborg, Anne-Lise Børresen-Dale, Bjørn Edwin, Åsmund Avdem Fretland, Krzysztof Grzyb, Mads H. Haugen, Gunhild M. Mælandsmo, Kjersti Flatmark

**Affiliations:** 1 Department of Tumor Biology, Institute for Cancer Research, Oslo University Hospital, Oslo, Norway.; 2 Institute of Clinical Medicine, University of Oslo, Oslo, Norway.; 3 Department of Oncology, Akershus University Hospital, Lørenskog, Norway.; 4 Department of Gastroenterological Surgery, Oslo University Hospital, Oslo, Norway.; 5 Department of Cancer Genetics, Institute for Cancer Research, Oslo University Hospital, Oslo, Norway.; 6 Department of Hepato-Pancreato-Biliary Surgery, Oslo University Hospital, Oslo, Norway.; 7 The Intervention Center, Oslo University Hospital, Oslo, Norway.; 8 Department of Pathology, Oslo University Hospital, Oslo, Norway.; 9 Institute for Medical Biology, Faculty of Health Sciences, UiT-The Arctic University of Norway, Tromsø, Norway.

## Abstract

Disease recurrence and drug resistance are major challenges in the clinical management of patients with colorectal cancer liver metastases (CLM), and because tumors are generally microsatellite stable (MSS), responses to immune therapies are poor. The mesenchymal phenotype is overrepresented in treatment-resistant cancers and is associated with an immunosuppressed microenvironment. The aim of this work was to molecularly identify and characterize a mesenchymal subgroup of MSS CLM to identify novel therapeutic approaches. We here generated a mesenchymal gene expression signature by analysis of resection specimens from 38 patients with CLM using ranked expression level of the epithelial-to-mesenchymal transition–related transcription factor *PRRX1*. Downstream pathway analysis based on the resulting gene signature was performed and independent, publicly available datasets were used to validate the findings. A subgroup comprising 16% of the analyzed CLM samples were classified as mesenchymal, or belonging to the *PRRX1*^high^ group. Analysis of the *PRRX1* signature genes revealed a distinct immunosuppressive phenotype with high expression of immune checkpoints *HAVCR2/TIM-3* and *VISTA,* in addition to the M2 macrophage marker *CD163.* The findings were convincingly validated in datasets from three external CLM cohorts. Upregulation of immune checkpoints *HAVCR2/TIM-3* and *VISTA* in the *PRRX1*^high^ subgroup is a novel finding, and suggests immune evasion beyond the PD-1/PD-L1 axis, which may contribute to poor response to PD-1/PD-L1–directed immune therapy in MSS colorectal cancer. Importantly, these checkpoints represent potential novel opportunities for immune-based therapy approaches in a subset of MSS CLM.

**Significance:**

CLM is an important cause of colorectal cancer mortality where the majority of patients have yet to benefit from immunotherapies. In this study of gene expression profiling analyses, we uncovered novel immune checkpoint targets in a subgroup of patients with MSS CLMs harboring a mesenchymal phenotype.

## Introduction

Metastatic colorectal cancer (mCRC) is a leading cause of cancer-related mortality, with colorectal cancer liver metastases (CLM) being the most common metastatic location (1). Although surgical resection is potentially curative for patients with CLM with limited disease burden, palliative chemotherapy is the main therapeutic option for most patients. Over the last decade, novel therapies targeting inhibitory immune checkpoints have increased survival of patients with metastatic cancer, but with exception of the small subgroup of microsatellite instable (MSI) tumors, colorectal cancer remains a nonimmunogenic cancer that responds poorly to immunotherapy (2).

Molecular classification of primary colorectal cancer has previously identified a subgroup characterized by upregulation of genes related to epithelial–mesenchymal transition (EMT; ref. 3), a phenotype that has been associated with drug resistance and an immunosuppressed microenvironment in colorectal cancer (4, 5) and in other cancers (6). In established mCRC, this mesenchymal phenotype has not been well characterized, and a more detailed understanding of this subgroup might reveal novel therapeutic opportunities. In this work, we aimed to investigate whether a mesenchymal phenotype subgroup could be identified by analysis of tumor samples from a cohort of patients with resectable CLM. An intrinsic molecular signature was generated on the basis of gene expression levels of the EMT-related transcription factor *PRRX1.* A CLM subgroup that exhibited high expression of *PRRX1* signature genes was identified and the findings were validated in independent CLM cohorts. The signature was associated with an immune-inflamed phenotype, harboring features of immune activation and suppression, and revealed potential novel targets for immune based therapies in patients with microsatellite stable (MSS) CLM.

## Materials and Methods

### Patients and CLM Samples

Metastatic tumor samples were collected at the time of CLM surgery from the first 71 patients enrolled in the Oslo-COMET trial (NCT01516710; refs. 7, 8). Of these, 33 cases were excluded from gene expression analysis for the following reasons: unresectable tumors (*n* = 2), benign lesions (*n* = 4; 2 hemangiomas, 1 focal nodular hyperplasia, and 1 fatty infiltration), no tissue for biobanking (*n* = 9), not analyzed (*n* = 1), inadequate tumor content (<10%; *n* = 9), and inadequate RNA quality (*n* = 8), leaving 38 CLM cases for analysis of which two metastatic lesions were available from six cases. The study was approved by the Regional Committee for Health and Research Ethics in Norway (2011/1285/REK Sør-Øst B), and written informed consent was required for participation. Median follow-up time was 66 months (95% confidence interval, 65–69) from CLM resection. Tumor tissue samples were snap frozen in liquid nitrogen immediately after resection and stored at −80°C. Two frozen sections per tumor sample were assessed for tumor content by the study pathologist (K. Grzyb) using routine diagnostic hematoxylin and eosin stains. Samples with tumor content 10%–100% (median 63%) were homogenized and aliquoted for further analysis. C-reactive protein (CRP) was measured in patient plasma as part of preoperative routine analysis. Date of death was obtained from the Norwegian National Registry.

### Mutation Analysis

Targeted next-generation sequencing has previously been described for this cohort (8). In brief, DNA was isolated by AllPrep DNA/RNA MiniKit (Qiagen) using the QiaCube system according to the manufacturer's instructions and quantified by NanoDrop ND-1000 (Thermo Fisher Scientific). Sequencing was conducted by using the Ion AmpliSeq Cancer Hotspot Panel (v2) covering mutational hotspots in 50 cancer-related genes and following the manufacturers’ protocol (Life Technologies). Data from the PGM runs were processed by The Torrent Suite Variant Caller using panel customized parameters as provided by Life Technologies and variants considered true passed quality control measures of minimum 500× coverage and at least 2% frequency.

### Microarray Gene Expression Analysis

Generation of microarray data has been described previously (8). Briefly, total RNA from fresh-frozen samples was isolated using TRIzol reagent (Invitrogen). All subsequent experimental procedures, including labeling, hybridization, and scanning, were processed according to the standard Affymetrix protocols associated with application of Agilent SurePrint G3 Human Gene Expression 8 × 60K arrays. The gene expression data were preprocessed with Agilent's Feature Extraction Software (v10.7.3.1) and quantile normalized and log_2_ transformed with R software. Probe sets representing unique genes were kept for analysis. When there were multiple probes per gene, the probe with the highest expression level was chosen.

### Construction of the *PRRX1* Signature

The variance in gene expression of the EMT-related transcription factors *ZEB1*, *ZEB2*, *SNAI1*, *SNAI2*, *TWIST1*, *TWIST2*, and *PRRX1* was analyzed to identify a factor with high variance which would enable stratification of the cohort by expression rank. Upon identifying *PRRX1* as the transcription factor exhibiting the highest variance, a quantile-based selection of the top and bottom 25% of the samples from unique patients was performed to form two groups (*n* = 10/group) for differential gene expression analysis. A significance analysis of microarrays (SAM) using J-Express software (http://www.molmine.com/JexpressMain.php) was performed contrasting these two groups and the resulting differentially expressed genes (DEG) with a FDR ≤ 0.001 were included in the mesenchymal signature. Hierarchical clustering was performed in R using the “heatmap.plus” package and using average linkage method with Euclidean distance. A per-sample signature score was generated by calculating the average log_2_ expression of all upregulated genes and used for subsequent correlation analysis with expression of key immune checkpoint genes. Subgroups of CLM samples were defined by major subbranches of the cluster dendogram and applied in subgroup statistical comparisons.

### Pathway Analysis and Estimation of Immune Cell Infiltration

Upregulated and downregulated genes with fold change data from the SAM analysis were uploaded into Ingenuity Pathway Analysis (IPA) software (Ingenuity Systems, www.ingenuity.com) for pathway enrichment analysis and functional annotation. Significance of each pathway and functional group was assessed by IPA using the Fisher’s exact tests (*P* ≤ 0.05). Upstream transcriptional regulation was predicted by IPA through the activation z-score statistic where the predicted regulatory relationships are associated with a direction of change that is either activating (z-score ≥ 2) or inhibiting (z-score ≤ −2). The functional annotation in IPA was run selecting “immune cells, liver and colorectal cancer cell lines” in the tissue/cell parameter setting to focus on the immunologic consequences of the mesenchymal phenotype. TIMER, a web-based open access resource (https://cistrome.shinyapps.io/timer/) was used to conduct deconvolution of infiltrating immune cell types based on transcriptomic data. TIMER estimated the abundances of six tumor-infiltrating cell populations including CD4^+^ T cells, CD8^+^ T cells, B cells, macrophages, neutrophils, and dendritic cells (DC).

### Reverse Phase Protein Array Analysis

Profiling of 295 cancer relevant proteins of which 63 were in a phosphorylated state (Supplementary Data S9) was performed at the reverse phase protein array (RPPA) core facility at MD Anderson Cancer Center (Houston, TX). Tissue from CLM (*n* = 30) was lysed then adjusted to 1 mg/mL concentration as assessed by bicinchonic acid assay and boiled with 1% SDS and 2-mercaptoethanol. Supernatants were manually diluted in five 2-fold serial dilutions with lysis buffer. The samples were spotted onto and immobilized on nitrocellulose-coated slides. The slides were probed with antibodies using a tyramide-based signal amplification approach and visualized by 3,3ʹ diaminobenzidine tetrahydrochloride (DAB) colometric reaction. Slides were scanned, analyzed, and spots quantitated using MicroVigene software (VigeneTech Inc.). Relative protein concentrations were derived from the supercurve for each sample by curve fitting using the R package “SuperCurve” (version 1.01). All the values were log_2_ transformed and median centered across each antibody. Differential protein expression analysis was performed as described for transcriptomic data.

### Validation of the *PRRX1* Signature

First, we validated that the *PRRX1* signature captures mesenchymal biology; second, the stratification performance of the signature and main biological findings were validated in three independent datasets. For the first approach, three public EMT/mesenchymal signature gene sets generated from meta-analyses across cancer types were curated (https://www.gsea-msigdb.org/gsea/msigdb/cards/HALLMARK_EPITHELIAL_MESENCHYMAL_TRANSITION.html; refs. 9, 10) and used to validate the presence of a subgroup with mesenchymal biology in the COMET cohort as identified by the *PRRX1* signature. From the meta-analysis published by Tan and colleagues (10), the gene subset exclusive to the mesenchymal phenotype was applied. The public gene sets are listed in Supplementary Data S5.

For external validation of the gene signature, the GSE41258, GSE10961, and GSE41568 datasets were downloaded from the NCBI Gene Expression Omnibus (GEO) using R's GEOquery package. GSE41258 consists of Rosetta/Merck Human RSTA Custom Affymetrix 2.0 microarray data from primary and mCRC samples, of which 21 CLM samples (11) were selected for our validation analyses. The GSE10961 dataset consisted of 18 CLM profiled on the Affymetrix Human Genome U133 Plus 2.0 Array (12). From GSE41568, we extracted 80 CLM (Affymetrix Human Genome U133 Plus 2.0 Array; ref. 13). For all three datasets, probes were matched to gene symbols using the annotation files provided by the manufacturer. The probe with highest expression was chosen if multiple probes matched to the same gene symbol. Hierarchical clustering was performed for all three datasets using the *PRRX1* signatures genes present in the external data which was array type dependent. From GSE10961 and GSE41568 expression data, *PRRX1* signature score per sample was calculated as described above.

### Statistical Analyses

Statistical analyses were conducted using the R Statistical Computing environment v.3.4.1 with exception of survival analysis where IBM SPSS statistics software was applied. Multiple groups were compared by one-way ANOVA. Unpaired two-tailed *t* test was used for pairwise comparisons. Pearson correlation was computed to correlate between *PRRX1* signature score and expression of immune markers. The Fisher’s exact test was applied for comparison of categorical clinical and mutational data between groups. A value of *P* ≤ 0.05 was considered statistically significant. Overall survival was calculated from CLM resection to date of death or censor date (December 31, 2017). HR was calculated using Cox proportional hazards analysis and was reported with 95% confidence interval.

### Data Availability

The data generated in this study are available upon request. The data that further support the findings of this study were obtained from NCBI GEO at GSE41568, GSE10961, and GSE41568.

## Results

### Patients

The 38 patients included in the analysis had a mean age of 66 years (min-max 46–81 years), 16 (42%) were women and 22 (58%) men. The primary tumor was located in the right colon in 10 cases (26%), in the left colon/rectum in 28 (74%). Twelve patients had received neoadjuvant chemotherapy. The frequency of colorectal cancer relevant mutations across the cohort has been published previously (8). To exclude systemic inflammation as a confounding factor contributing to the immune profile of the *PRRX1*^high^ group, serum levels of CRP at the time of CLM resection were evaluated. Generally, the levels were low (median = 2.8 mg/L; min-max 0.6–45 mg/L; where nine cases had CRP levels above the 5 mg/L threshold value). All included patients had MSS tumors (14).

### The *PRRX1* Signature and Cohort Subgroups

**FIGURE 1:**
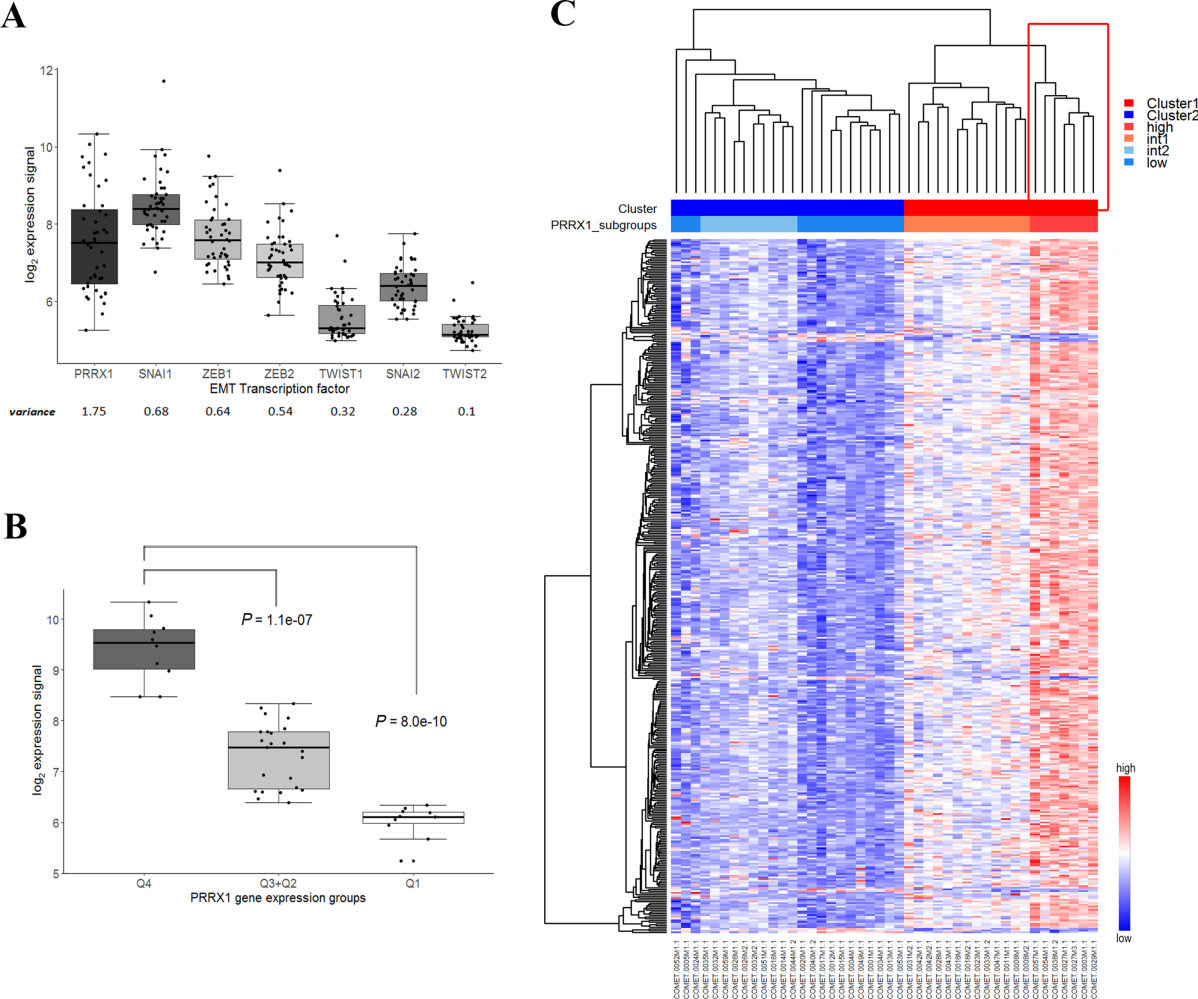
*PRRX1* gene and signature expression. **A,** Analysis of EMT-related transcription factor gene expression range in the COMET CLM cohort. Underlying bar displays variance per gene. **B,***PRRX1* gene expression in quartile-based selected groups. The samples were categorized into three groups according to levels of *PRRX1* expression: Q1 (low): 0%–25% quartile, Q2+Q3 (inter-medium): 25%–75% quartile, and Q4 (high): 75%–100% quartile. Significant differences in pairwise comparisons where Q4 was set as reference (*t* test). **C,** Heatmap and hierarchical clustering of the CLM samples based on the 405 *PRRX1* DEGs. Red-blue scale reflects log_2_ expression (range, 4.3–16.3). The rows above the heatmap depict main clusters (blue and red as shown on the top “Cluster” row) and subgroups (blue, light-blue, orange-red, and red as shown on the second “PRRX1_subgroups” row) defined by dendrogram subbranches. Seven samples fall into a distinct *PRRX1*^high^ cluster (red box).

The *PRRX1* transcription factor displayed the highest expression variance of the investigated EMT-related transcription factors with a continuous distribution across the cohort between extreme high/low values (Fig. 1A) and was applied to distinguish high and low expression subgroups for differential expression analysis assuming enrichment of the mesenchymal phenotype in the high expression subgroup. When comparing the high *PRRX1* gene expression group (Q4 = top 25% of the samples) with the low *PRRX1* expressing group (Q1 = bottom 25% of the samples; Fig. 1B), 405 DEGs were identified (FDR of <0.1%; Supplementary Data S1). Hierarchical clustering using the *PRRX1* DEGs split the samples into two distinct clusters defined by two major subbranches of the dendrogram (Fig. 1C). Cluster 1 (right major subbranch) showed higher expression of the *PRRX1* signature genes (more mesenchymal) and Cluster 2 (left major subbranch) showed low expression (more epithelial). Each cluster showed further subbranching into two subclusters, reflecting an overall distribution of *PRRX1*^high^ and inverse *PRRX1*^low^ phenotypes at each end of a spectrum of intermediate phenotypes (Fig. 1C). Four subgroups were defined for downstream analyses based on the dendogram: *PRRX1*^high^ (*n* = 7), *PRRX1*^int1^ (*n* = 13), *PRRX1*^int2^ (*n* = 10), and *PRRX1*^low^ (*n* = 14; Supplementary Data S2). Three outlier samples were merged into the *PRRX1*^low^ subgroup as their signature scores matched the score range of this subgroup. The cohort included CLM pairs from 6 patients and no pairs were split into separate subgroups defined by the clustering. The *PRRX1*^high^ subgroup, constituting 16% of the cohort samples, displayed the highest expression of signature genes (mesenchymal phenotype; Fig. 1C) and was the focus group of downstream statistical comparisons.

### The *PRRX1* Signature Genes Define a Mesenchymal and Immune Phenotype

Of the 405 DEGs in the *PRRX1* signature, 393 were upregulated and 12 downregulated. IPA identified “Hepatic fibrosis” (*P* = 3.1E-26) and “Regulation of EMT pathway” (*P* = 3.2E-09) among the top 10 enriched canonical pathways (Table 1). In addition, immune response pathways such as “Leukocyte extravasation signaling” (*P* = 8.4E-09), “Dendritic cell maturation” (*P* = 5.9E-11), “Th2 pathway” (*P* = 2.7E-09), and “Th1 pathway” (*P* = 2.7E-08) were among the highest ranked pathways, suggesting immune cell recruitment and activation. The Upstream Regulator analysis (IPA) predicted *TGFB1*, *SMARCA4*, *HIF1A*, *TNF,* and *IFNG* as top activated regulators while *SPDEF* and *FBN1* were predicted to be inhibited regulators (negative activation scores; Supplementary Data S3).

**TABLE 1:** IPA canonical pathway analysis (rank: *P*-value)

Canonical pathways	*P*-value	z-score	Molecules
Hepatic fibrosis/Hepatic stellate Cell activation	3.1E-26	NaN	COL8A2,CCR5,ICAM1,CTGF,COL8A1,COL10A1,COL4A2,COL15A1,COL5A1, COL1A2,COL6A1,TGFB1,PDGFRA,TIMP2,PDGFRB,COL5A2,VCAM1, COL4A1,COL6A2,FGFR1,COL12A1,VEGFC,IGFBP5,MMP2,NFKB2,COL1A1, COL5A3,LY96,COL13A1,ACTA2,COL6A3,TGFB3,EDNRA,IL1B,A2M,COL3A1
GP6 signaling pathway	5.9E-15	4.7	COL8A2,COL5A2,COL4A1,COL6A2,COL12A1,FGFR1,LAMA2,COL8A1, COL10A1,ITPR1,COL4A2,COL15A1,COL5A1,COL1A2,COL1A1,COL5A3, COL6A1,COL13A1,COL6A3,AKT3,LCP2,COL3A1
Dendritic cell maturation	5.9E-11	4.4	FCGR2C,ICAM1,TYROBP,FGFR1,HLA-DQA1,COL10A1,NFKB2,FCGR2B, TLR2,COL1A2,COL1A1,COL5A3,HLA-DMA,HLA-DRB3,HLA-DMB, HLA-DRA,AKT3,IL1B,FCGR3A/FCGR3B,FCGR1B,COL3A1
Th2 pathway	2.7E-09	0.7	CCR1,RUNX3,CCR5,NOTCH3,ICAM1,CXCR4,FGFR1,HLA-DQA1,TGFB1, HLA-DMA,HLA-DRB3,HLA-DMB,HLA-DRA,VAV1,HLA-DQA2,HLA-DPB1, HLA-DPA1
Regulation of the EMT pathway	3.2E-09	NaN	LOX,TCF4,NOTCH3,SNAI2,FGFR1,TWIST1,MMP2,NFKB2,ZEB1,WNT2, PYGO1,TGFB1,ZEB2,TGFB3,MRAS,AKT3,LEF1,PDGFRB,WNT5A
Th1 and Th2 activation pathway	1.1E-08	NaN	CCR1,RUNX3,CCR5,NOTCH3,ICAM1,CXCR4,FGFR1,HAVCR2, HLA-DQA1,HLA-DMA,TGFB1,HLA-DRB3,HLA-DMB,HLA-DRA,VAV1, HLA-DQA2,HLA-DPB1,HLA-DPA1
Leukocyte extravasation signaling	1.6E-08	2.5	VCAM1,ICAM1,CXCR4,FGFR1,THY1,MMP2,RHOH,NCF1,ITGAM,EDIL3, ACTA2,JAM3,NCF2,CYBB,ITGA1,MMP11,VAV1,DLC1,TIMP2
Th1 pathway	2.7E-08	3	RUNX3,CCR5,NOTCH3,ICAM1,FGFR1,HAVCR2,HLA-DQA1,HLA-DMA, HLA-DRB3,HLA-DRA,HLA-DMB,VAV1,HLA-DQA2,HLA-DPB1,HLA-DPA1
Neuroinflammation signaling pathway	8.1E-08	4.5	VCAM1,NOX4,ICAM1,TYROBP,FGFR1,SLC1A3, HLA-DQA1,NFKB2,IRAK3,CSF1R,TLR2,HLA-DMA,TGFB1, HLA-DMB,NCF2,TLR1,HLA-DRA,CYBB,TGFB3,TLR7,AKT3,IL1B
Colorectal cancer metastasis signaling	2.2E-07	3.3	TCF4,FGFR1,VEGFC,MMP2,NFKB2,RHOH,WNT2,TLR2,GNB4,GNG11, TGFB1,TLR1,MRAS,TGFB3,TLR7,AKT3,LEF1,MMP11,WNT5A

The top upregulated genes (*LUM*, *CYR61*, *CCL18*, *AEBP1*, *SULF1,* and *INHBA,* fold change (FC) = 14.6–9.8) in the *PRRX1* DEG list were briefly explored and collectively they support a relationship between the mesenchymal phenotype and enrichment of immune responses. These genes are reported as mediators of inflammation and immune modulatory activities, in addition to promoting induction of EMT (15–20). Among the 12 downregulated genes (FC range from −1.5 to −2.9) was *TRAP1*, a member of the *HSP90* protein family, which is associated with induction of EMT in ovarian cancer (21). Three zinc finger protein genes and *SLC39A4* (*ZIP4*) which all have been associated with zinc homeostasis were also among the downregulated genes.

**FIGURE 2:**
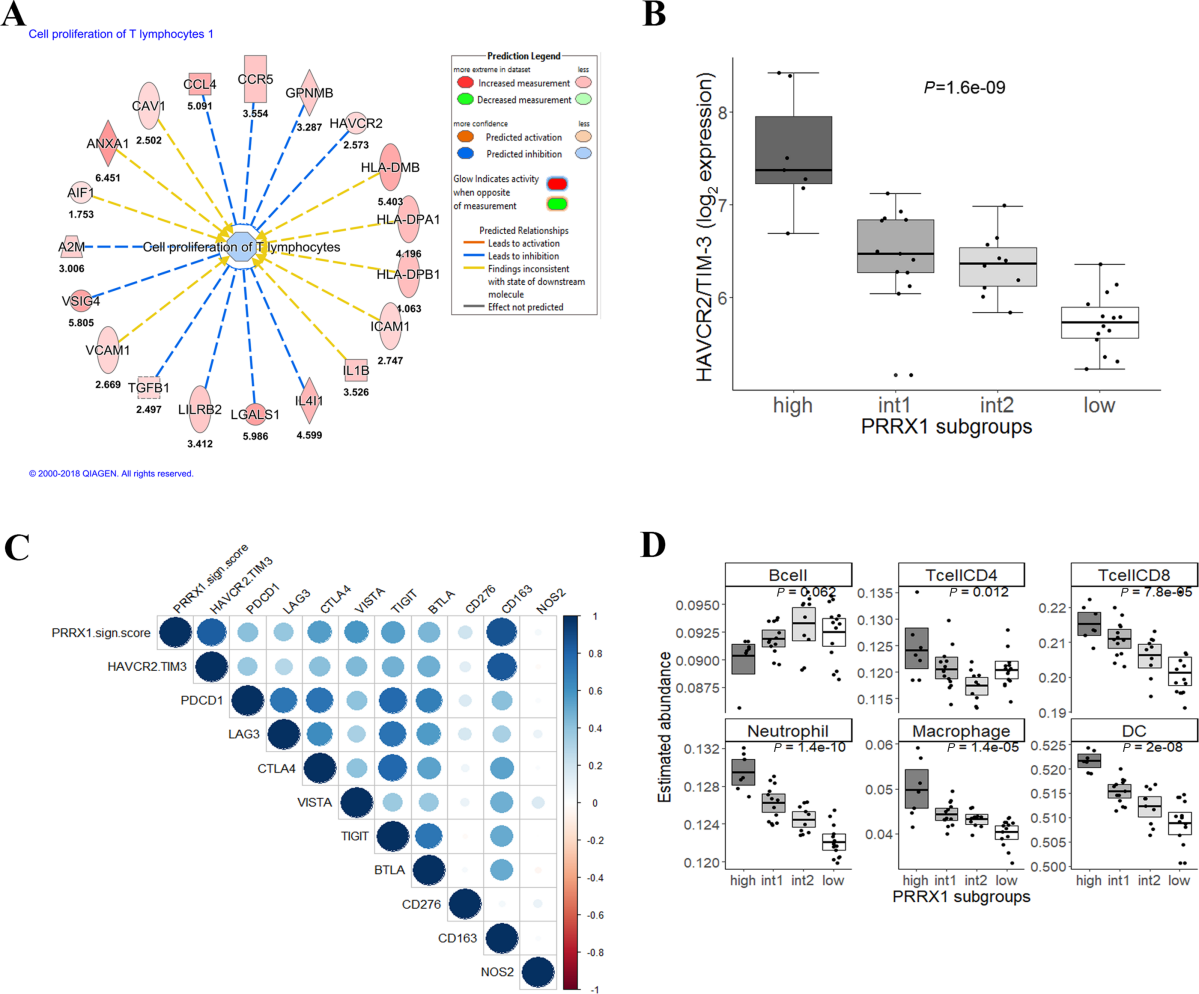
Immunologic features associated with the *PRRX1* signature. **A,** “Cell proliferation of T lymphocytes” was a significant biological function to be associated with the *PRRX1* signature genes. The function was predicted to be inhibited in the *PRRX1*^high^ subgroup. The blue lines indicate candidate upregulated genes associated with inhibition of proliferation of T cells. **B,** Gene expression of *HAVCR2/TIM-3* across *PRRX1* subgroups. Statistical significance was assessed by ANOVA in a multiple group comparison. **C,** Correlation matrix between the *PRRX1* signature score and expression of key immune checkpoint genes and M2 (*CD163*) and M1 (*NOS2*) macrophage markers. **D,** Boxplots of immune cell abundances. Estimated abundance of immune cells based on gene expression data using the TIMER application. Abundance of B cells, DCs, macrophages, neutrophils, CD4^+^ T cells, and CD8^+^ T cells in CLM samples estimated according to *PRRX1* subgroup. Statistical significance was assessed by ANOVA in a multi-group comparison.

Immunologic features associated with the *PRRX1* DEGs were further analyzed by gene ontology enrichment analyses in IPA. The most significant biological functions found to be associated with the gene list were related to movement and adhesion of immune cells. Notably, “Proliferation of Immune Cells” (*P* = 3.8E-08) was predicted to be inhibited and more specifically, “Cell proliferation of T lymphocytes” (*P* = 5.3E-07) had a negative z-score (inhibited; Supplementary Data S4). Candidate genes contributing to this predicted inhibitory status are depicted in Fig 2A, among them *TGFβ*, *LGALS1,* and *CCR5*. Notably, the inhibitory immune checkpoint receptor gene *HAVCR2*, also known as *TIM-3* (FC = 2.6), was among the genes functionally assigned to “Cell Proliferation of T lymphocytes.”

### The *PRRX1* Signature—Associations with Key Immune Checkpoint Genes and Infiltrating Immune Cells

When analyzing correlations between *PRRX1* signature score and gene expression of key immune checkpoint molecules, a strong positive correlation was observed with expression of *HAVCR2/TIM-3* (Pearson Corr = 0.83, *P* = 4.5E-12; Fig. 2B). Correlations with expression of other immune checkpoint genes were in the range of 0.58–0.21, (*P* < 0.05 with the exception of *CD276* with *P* value = 0.18) where notably, *VISTA* (Pearson Corr = 0.58, *P* = 3.6E-05), *CTLA4* (Pearson Corr = 0.55, *P* = 1.0E-04), and *TIGIT* (Pearson Corr = 0.54, *P* = 1.4E-04) ranked highest (Fig. 2C). Furthermore, a strong positive correlation was found between the signature score and the M2 macrophage marker *CD163* (Pearson Corr = 0.86, *P* = 4.6E-14; Fig. 2C).

The distinct immune profile of the *PRRX1*^high^ subgroup was further revealed by gene expression–based deconvolution analysis (TIMER), which identified significant differences in the relative abundance of immune cell subsets when comparing the *PRRX1*-expression subgroups (Fig. 2D). The *PRRX1*^high^ subgroup exhibited enrichment of neutrophils, DCs, macrophages, CD8^+^ T cells and CD4^+^ T cells, but lower abundance of B cells in a multi-group comparison. The most significant differences were observed for neutrophils (*P* = 1.4E-10) and DC (*P* = 2.0E-08; Fig. 2D).

### Validation of the *PRRX1* Signature by Comparison with Other EMT/Mesenchymal Signatures

**FIGURE 3:**
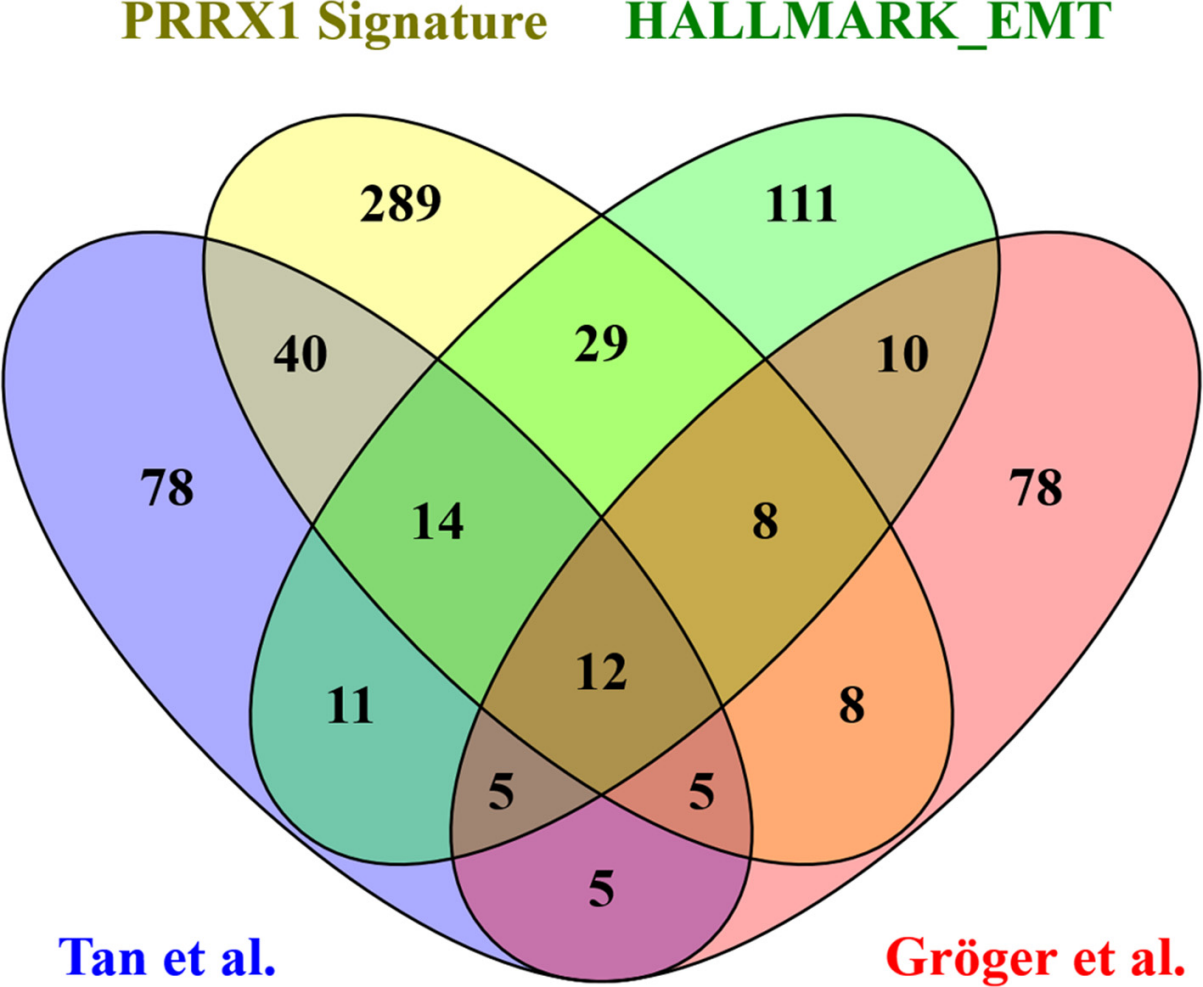
Venn diagram of signature genes. Overlapping sets of genes between the *PRRX1* signature and three public EMT core signatures based on meta-analyses [References: Broad Institute (9, 10); see Supplementary Data S5]. The diagram was made using the online tool Venny (http://bioinfogp.cnb.csic.es/tools/venny/index.html).

Three public EMT/mesenchymal signatures generated independently from meta-analyses across cancer types (Table 2; Supplementary Data S5) were used to validate that the *PRRX1* signature captures a subgroup with mesenchymal biology. Overlapping genes between the three public signatures and the *PRRX1* signature are depicted in Fig. 3. Twelve genes were common for all four gene sets (*CDH11*, *DCN*, *EMP3*, *FBN1*, *FSTL1*, *LOX*, *MMP2*, *PMP22*, *SPOCK1*, *TAGLN*, *VCAN*, and *VIM*). Five genes were overlapping in the three meta-analyses but absent from the *PRRX1* gene list (*CDH2*, *FBLN1*, *FN1*, *MYL9*, and *PTX3*). The *PRRX1* gene was present in the Hallmark_EMT gene set but was absent from the two other public gene signatures. When clustering analysis was performed, all signatures identified a mesenchymal subgroup in the cohort that largely overlapped with the *PRRX1*^high^ subgroup (Supplementary Fig. S1A–S1D).

**TABLE 2:** Public and cohort-intrinsic EMT/MES signatures

Gene signature	No. of genes	Cancer type	Source
HALLMARK_EPITHELIAL_MESENCHYMAL_TRANSITION	200	Meta-analysis	Broad Institute
Generic EMT signature subset: tumor_MES	170	Meta-analysis	Tan *et al*., EMBO, 2014 (10)
EMT core signature	131	Meta-analysis	Gröger *et al*., PLosOne, 2012 (9)
*PRRX1* signature	405	CRC liver metastases	COMET

### Validation of the *PRRX1* Signature in Independent Datasets

The discriminatory power of the *PRRX1* signature was further validated in three independent cohorts of CLM patient samples (Supplementary Fig. S2A, S2B, and S2E). Of the 405 *PRRX1* signature genes, expression data for 304 genes were present in GSE41258 and 370 genes in both GSE10961 and GSE41568 datasets (array type dependent). The *PRRX1* signature revealed cohort stratification in all three datasets by binary partitioning of samples into Cluster 1 and Cluster 2 subgroups. The Cluster 1 subgroup with higher *PRRX1* signature expression constituted 33% (GSE41258), 33% (GSE10961), and 16% (GSE41568) of the samples, respectively, which is in line with the *PRRX1*^high^ subgroup of 16% in our cohort.

The *PRRX1* signature score was calculated for samples in the GSE10961 and GSE41568 datasets to analyze the correlation between the signature score and expression of immune checkpoint molecules (Supplementary Fig. S2C and S2F). Strikingly, positive correlations were observed with *HAVCR2/TIM-3* (Pearson Corr = 0.75, *P* = 3.5E-04), *VISTA* (Pearson Corr = 0.66, *P* = 3.0E-03), *CTLA4* (Pearson Corr = 0.48, *P* = 4.2E-02), and *CD163* (Pearson Corr = 0.63, *P* = 4.7E-03) when analyzing the GSE10961 data, reflecting our findings exactly. Data from the larger public cohort (GSE41568) further confirmed the correlation with *HAVCR2*/*TIM-3* (Pearson Corr = 0.78, *P* = 2.2E-16), *VISTA* (Pearson Corr = 0.55, *P* = 9.5E-08), and *CD163* (Pearson Corr = 0.66, *P* = 2.9E-11).

When Cluster 1 and 2 were compared using TIMER analysis in GSE10961 and GSE41568, similar enrichment of immune cell subsets was observed in Cluster 1 as in the *PRRX1*^high^ subgroup in the COMET cohort (Supplementary Fig. S2D and S2G).

### Validation of the *PRRX1* Signature by Protein Expression Data

**FIGURE 4:**
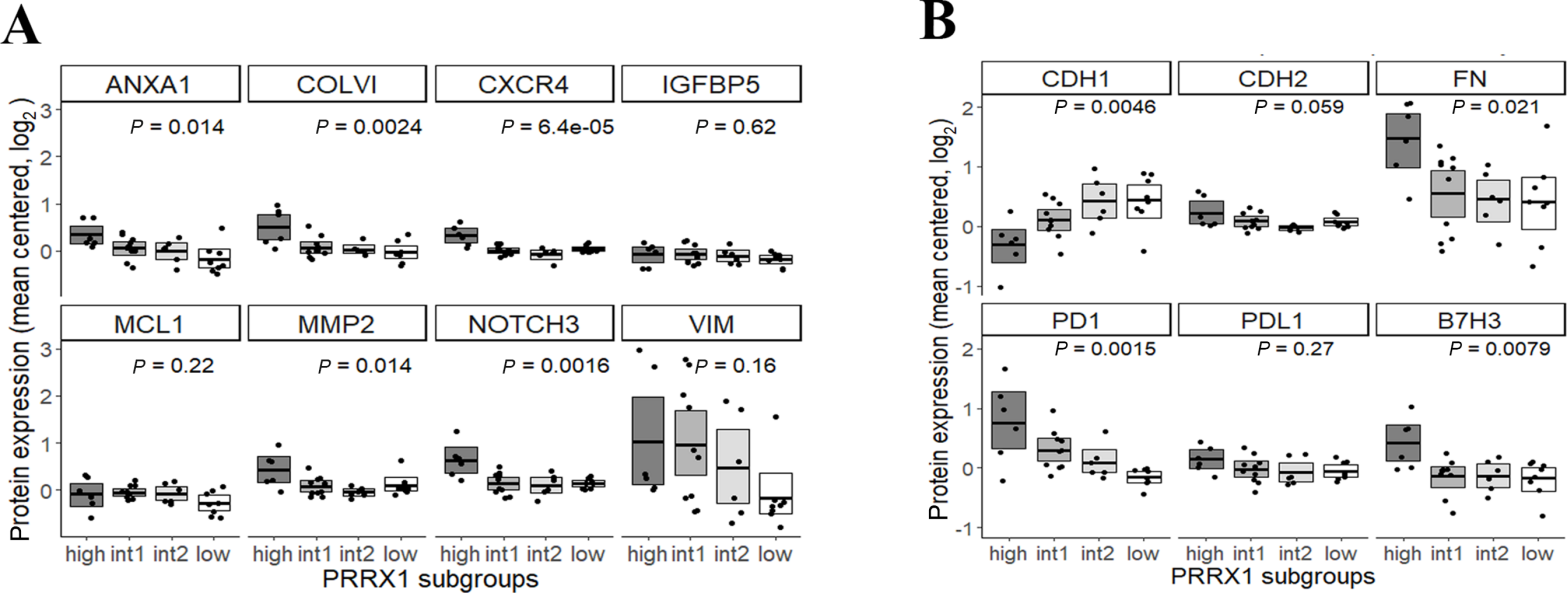
Boxplots of protein quantification. **A,** Protein expression of *PRRX1* signature genes. Of the eight genes with protein data, *PRRX1* subgroup specific expression is significant for five proteins, all showing a higher expression in the *PRRX1*^high^ subgroup. **B,** Protein quantification by RPPA of EMT- (top) and immune-related proteins (bottom). Statistical significance was assessed by ANOVA in a multi-group comparison.

Limited overlap existed between RPPA antibody targets and *PRRX1* signature genes; reflecting the available version of RPPA. Eight *PRRX1* signature genes were represented by RPPA data, of which five proteins showed significant variance and higher expression in the *PRRX1*^high^ subgroup in accordance with gene expression analysis (Fig. 4A). Expression of classical markers of EMT, such as E-cadherin (CDH1), N-cadherin (CDH2), and fibronectin (FN), in addition to the immune checkpoint molecules PD-1/PD-L1 and B7H3 further supported the EMT/MES and immunologic phenotype of the *PRRX1*^high^ subgroup (Fig. 4B).

### Associations between the *PRRX1*^high^ Signature and Clinicopathologic Features and Long-term Outcome

All patients in the *PRRX1*^high^ subgroup were women (*P* = 0.004), and the *PRRX1*^high^ group was associated with right-sided primary colorectal cancer (*P* = 0.03; Supplementary Data S6–S8; Supplementary Fig. S3). Comparing mutational profiles obtained by targeted sequencing, the *PRRX1*^high^ subgroup contained more cases with *TP53* mutations (*P* = 0.005). Of 38 patients, 20 (53%) died during follow-up period. There were no significant differences in overall survival between the *PRRX1*^high^ subgroup and the remaining cohort [HR, 1.05 (0.31–3.61), *P* = 0.94].

## Discussion

In this work, taking an unbiased approach, the expression of a number of EMT-related transcription factors was analyzed and *PRRX1* was identified as the factor exhibiting the largest expression variance in our cohort of CLM cases. Differential gene expression analysis was then applied to identify *PRRX1* coexpressed genes which were used to generate the *PRRX1* signature and define the *PRRX1*^high^ subgroup. Importantly, the signature was validated by applying public pan cancer EMT signatures to our data (reidentifying the *PRRX1*^high^ subgroup), by applying the *PRRX1* signature to three independent CLM datasets, and by protein expression analysis, supporting the reproducibility and relevance of the signature. Not only was a *PRRX1*^high^ subgroup validated in all the investigated CLM datasets; the identified subgroup exhibited high expression of the same checkpoint molecules and a similar enrichment of immune cells across the analyzed datasets. The signature validation lends confidence in the analytic approach and supports the clinical validity of the *PRRX1* signature.

The mesenchymal phenotype is a fundamental feature of primary colorectal cancer classification, and is strongly associated with poor prognosis, tumor recurrence, and drug resistance (5, 22, 23). The current work provides evidence that the mesenchymal phenotype can also be detected in the metastatic setting in CLM samples. Acquisition of a mesenchymal phenotype has been associated with T-cell dysfunction through increased expression of checkpoint molecules (24–26), and a pan-cancer EMT signature was previously suggested as a tool to select patients that might benefit from immune checkpoint inhibition (26). Therefore, identification of an equivalent subgroup in mCRC could be of clinical relevance with respect to new target discovery within immune checkpoint–directed therapy which could particularly benefit patients that have developed resistance to chemotherapy.

Functional annotation of the *PRRX1* signature genes revealed a strong association with immune-related genes and processes, displaying both antitumor (Th1) and tumor-tolerant (Th2) responses, which was consistent with the predicted enrichment of immune cells in the *PRRX1*^high^ subgroup. Applying the TIMER algorithm, recruitment of several distinct immune cell types was estimated, with a higher relative abundance of DCs, neutrophils, macrophages, and T cells (CD4^+^ and CD8^+^) in the *PRRX1*^high^ subgroup. Balancing the immune activation driven by *IFNγ* signaling, TGFβ was predicted as upstream regulator of DEGs representing immune suppressive molecules upregulated in the *PRRX1*^high^ subgroup. Interestingly, the *PRRX1* signature was positively correlated with expression of *CD163,* an established marker of the immune suppressive M2-polarized macrophage population, which has been associated with induction of EMT (27, 28). Despite the apparent immune suppression, the evidence of a preexisting immune activation is of importance as it could in principle be reactivated by appropriate immune modulating therapies.

Tumor immune escape from an ongoing immune activation can upregulate immune checkpoint expression. Targeting immune tolerance via coinhibitory checkpoint molecules to restore cytotoxic T-cell function forms the basis of current immune therapies. The immune profile associated with the *PRRX1* signature suggested the presence of dysfunctional T cells with checkpoint molecule involvement, in addition to an imbalance between protumor and antitumor immune cells as mentioned above. The mechanistic link between the *PRRX1*-driven mesenchymal phenotype and checkpoint molecule upregulation remains to be identified as this cannot be established from our gene expression dataset alone. Methods such as spatial transcriptomics and single-cell analysis would be logical steps to validate the signature and further investigate signature contribution from both tumor and stromal components. However, the strong correlation between the *PRRX1* signature and expression of immune checkpoint molecules is in striking accordance with the findings of a recent study of patients with metastatic gastric adenocarcinoma where the mesenchymal-like tumor subgroup had high expression of *HAVCR2/TIM-3* and *VISTA* (29). Furthermore, *TIM-3* gene expression was identified as a top contributing factor to the distinct clustering of an EMT-high colorectal subgroup in a pan-cancer study (30). The clinical significance of identifying *HAVCR2/TIM-3* and *VISTA* associated with the *PRRX1* signature score is thus dual. These checkpoints represent potential resistance markers to the widely applied immune therapies targeting the PD-1/PD-L1 axis, and their high expression could contribute to explain inherent resistance to this approach in MSS mCRC. HAVCR2/TIM-3 is an emerging clinical target in the cancer immune landscape along with VISTA. *HAVCR2/TIM-3* has been reported to be upregulated in response to PD-1 blockade in various cancer models (31, 32), and overexpression of *HAVCR2/TIM-3* and *VISTA* has been associated with lack of response to anti-PD-1/PD-L1–based therapies (33). HAVCR2/TIM-3 and VISTA therefore represent alternative candidate immune targets that based on our results could be explored in patients of the *PRRX1*^high^ subgroup. There are currently a number of signal-seeking early phase clinical trials evaluating anti-TIM3 antibodies in advanced cancer patients as monotherapy or in combination (e.g., NCT03489343, NCT03680508, and NCT02817633). Similarly, VISTA, which also has entered early trial phase (e.g., NCT04475523, NCT04564417), is a particularly interesting cotarget due to its expression on both exhausted T cells and infiltrating suppressive myeloid cells which may differentiate into tumor-associated macrophages (34). Targeting VISTA may potentially reduce populations of infiltrated immune suppressive cells which may be required for restoring T-cell effecter function by anti-HAVCR2/TIM-3 blockade.

An important limitation of this work is related to a limited sample size, but this is partly compensated by the validation analyses. Also, the selective analysis of resected liver metastases in the COMET trial may have implications for the representativeness of the findings. However, for ethical reasons, larger series of biopsy samples from nonresectable cases are not available, leaving researchers to base analyses on resected samples. In addition, the analyses were performed using transcriptional data generated from bulk tissue, limiting analysis of which cell type has contributed to a specific profile to the application of deconvolution methods, thereby limiting interpretation regarding underlying biological mechanisms. In future studies, analyses including all *PRRX1* subgroups could be extended to include more advanced antibody-based cytometry analyses using live cell suspensions or intact tissues to gain further resolution of functional immune subtypes present in the CLM mesenchymal phenotype. Furthermore, multi-marker detection methods could be used to validate protein expression and place TIM-3 and VISTA expression into context of relevant immune markers for a more comprehensive understanding.

Although the starting point for this work was an explorative study of limited sample size, the results regarding checkpoint molecule expression and predicted immune cell enrichment in the *PRRX1*^high^ subgroup were convincingly and reproducibly validated in three independent CLM cohorts (totaling 119 cases). The uncovered biology provides rationale for incorporating immune modulating therapy tailored to a specific CLM patient subgroup defined by the *PRRX1* signature and suggests further exploration of the novel immune checkpoints HAVCR2/TIM-3 and VISTA in MSS mCRC. The *PRRX1* signature may help identify patients with CLM likely to benefit from immune-based therapies directed at these targets and points to an opportunity for expanding the use of immune therapy strategies to patients with mCRC beyond the MSI subgroup. Our next goal will be to further develop the *PRRX1* signature for clinical utility as a predictive biomarker, using feature reduction tools and test in biopsy samples from our ongoing immune therapy trial in MSS mCRC (NCT03388190).

## Supplementary Material

Supplementary Figures S1-3 Figure S1. Gene expression heatmaps. Identification of an EMT/MES subgroup.
Figure S2. Validation of the PRRX1 signature in independent CLM data sets.
Figure S3. CRP measure in patient blood samples analyzed across PRRX1 subgroups.

Supplementary File S1 Supplementary File S1 Data S1-S9
