# Supplementary material for: A PRRX1 Signature Identifies TIM-3 and VISTA as Potential Immune Checkpoint Targets in a Subgroup of Microsatellite Stable Colorectal Cancer Liver Metastases
Source: Cancer Res Commun. 2023 Feb 9;3(2):235–44. doi: 10.1158/2767-9764.CRC-22-0295 (PMC10035516; doi:10.1158/2767-9764.CRC-22-0295)
Supplement: Supplementary Figures S1-3 — Figure S1. Gene expression heatmaps. Identification of an EMT/MES subgroup. Figure S2. Validation of the PRRX1 signature in independent CLM data sets. Figure S3. CRP measure in patient blood samples analyzed across PRRX1 subgroups. [file crc-22-0295-s01.pptx]

## Slide 1
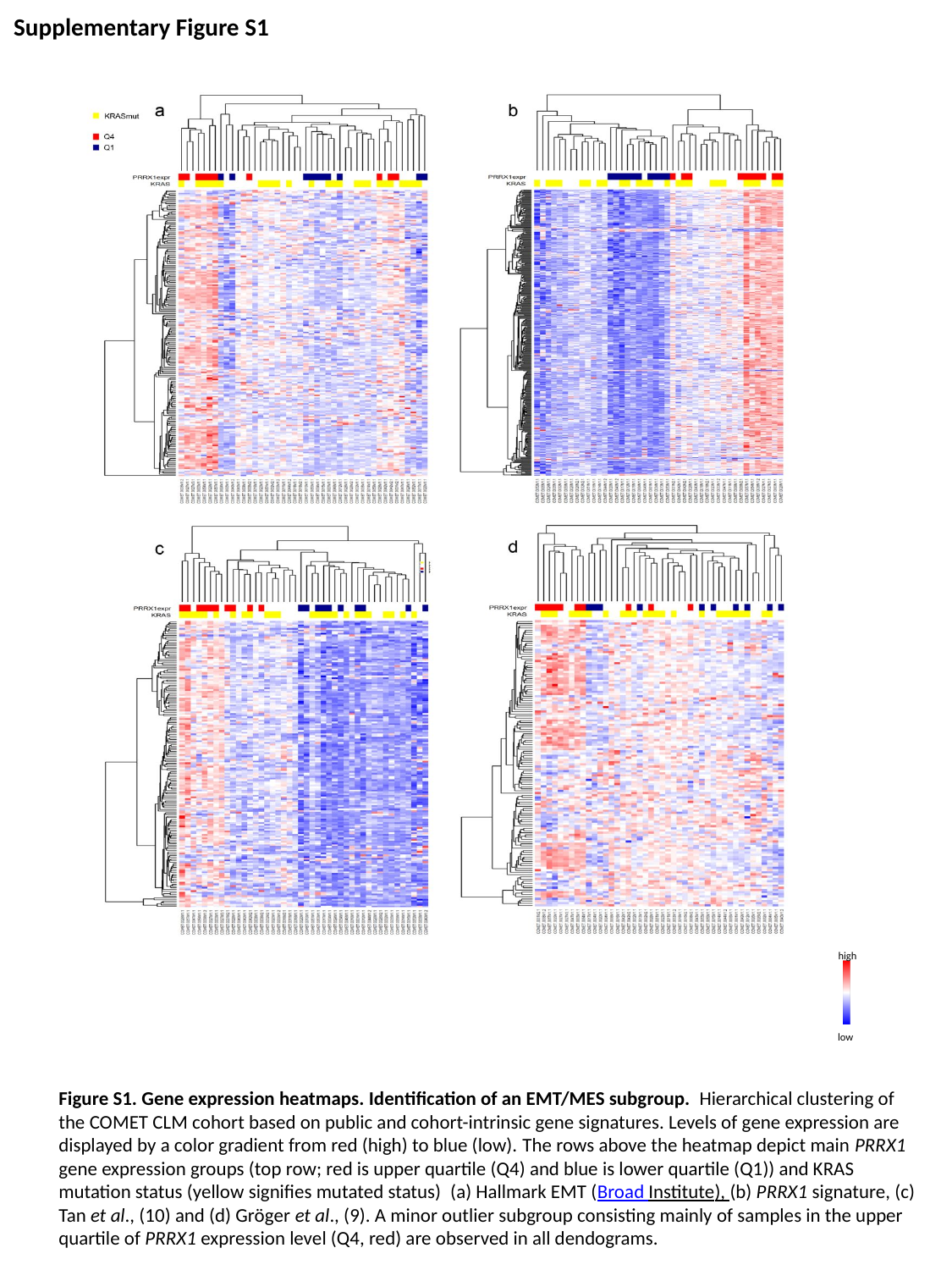

Supplementary Figure S1
high
low
Figure S1. Gene expression heatmaps. Identification of an EMT/MES subgroup. Hierarchical clustering of the COMET CLM cohort based on public and cohort-intrinsic gene signatures. Levels of gene expression are displayed by a color gradient from red (high) to blue (low). The rows above the heatmap depict main PRRX1 gene expression groups (top row; red is upper quartile (Q4) and blue is lower quartile (Q1)) and KRAS mutation status (yellow signifies mutated status) (a) Hallmark EMT (Broad Institute), (b) PRRX1 signature, (c) Tan et al., (10) and (d) Gröger et al., (9). A minor outlier subgroup consisting mainly of samples in the upper quartile of PRRX1 expression level (Q4, red) are observed in all dendograms.

## Slide 2
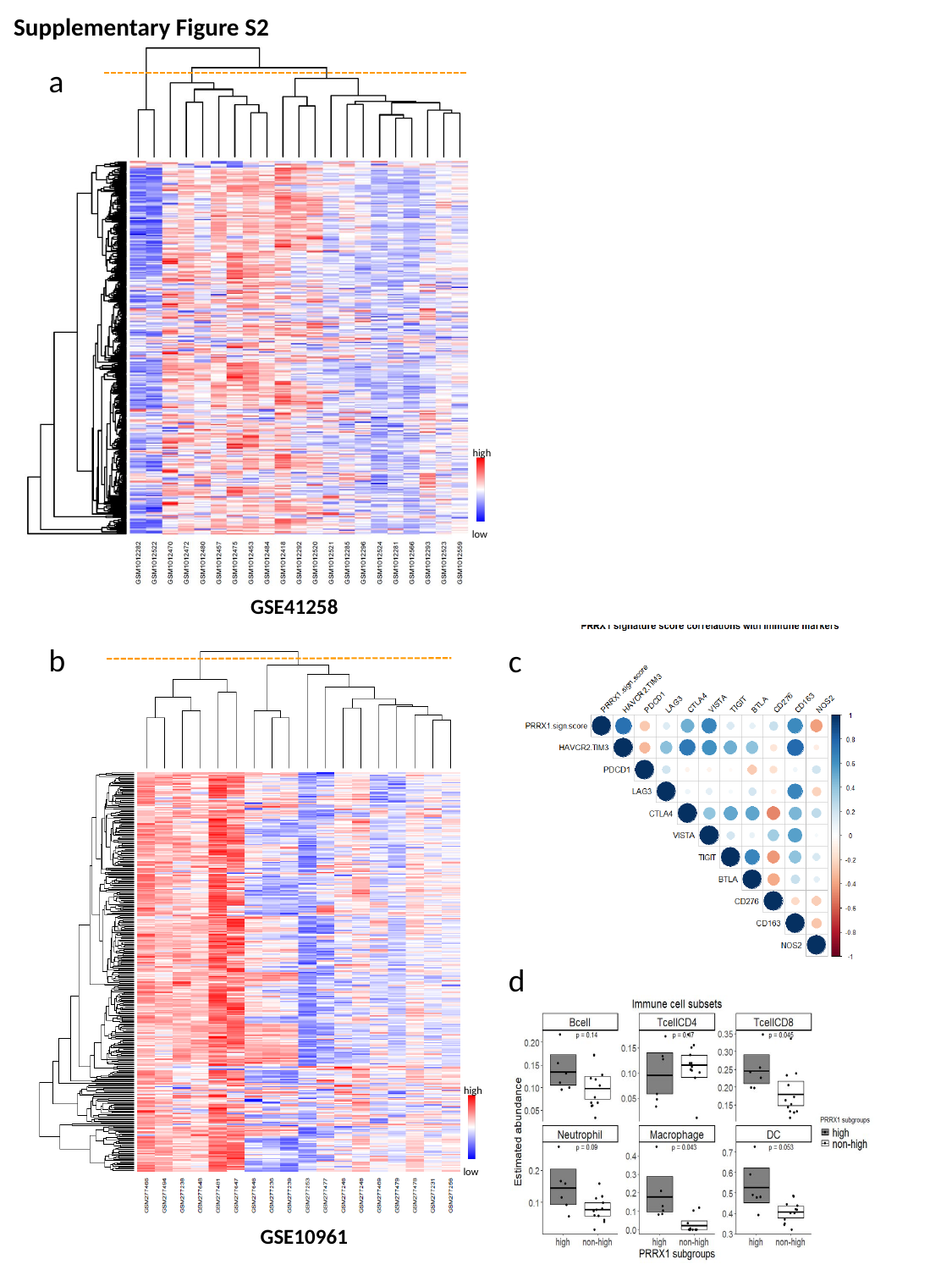

Supplementary Figure S2
GSE41258
a
high
low
b
c
GSE10961
d
high
low

## Slide 3
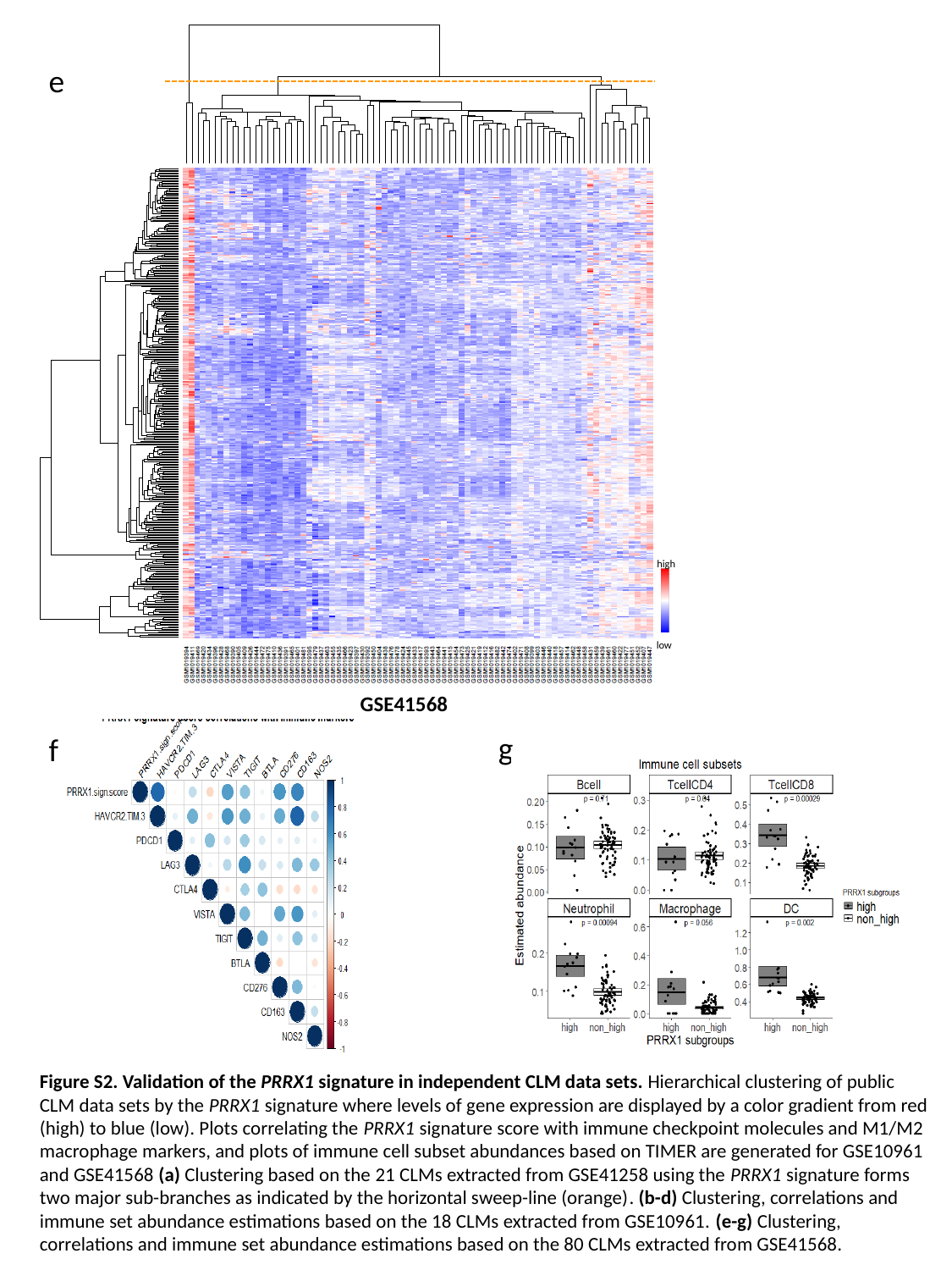

c
GSE41568
e
high
low
g
f
Figure S2. Validation of the PRRX1 signature in independent CLM data sets. Hierarchical clustering of public CLM data sets by the PRRX1 signature where levels of gene expression are displayed by a color gradient from red (high) to blue (low). Plots correlating the PRRX1 signature score with immune checkpoint molecules and M1/M2 macrophage markers, and plots of immune cell subset abundances based on TIMER are generated for GSE10961 and GSE41568 (a) Clustering based on the 21 CLMs extracted from GSE41258 using the PRRX1 signature forms two major sub-branches as indicated by the horizontal sweep-line (orange). (b-d) Clustering, correlations and immune set abundance estimations based on the 18 CLMs extracted from GSE10961. (e-g) Clustering, correlations and immune set abundance estimations based on the 80 CLMs extracted from GSE41568.

## Slide 4
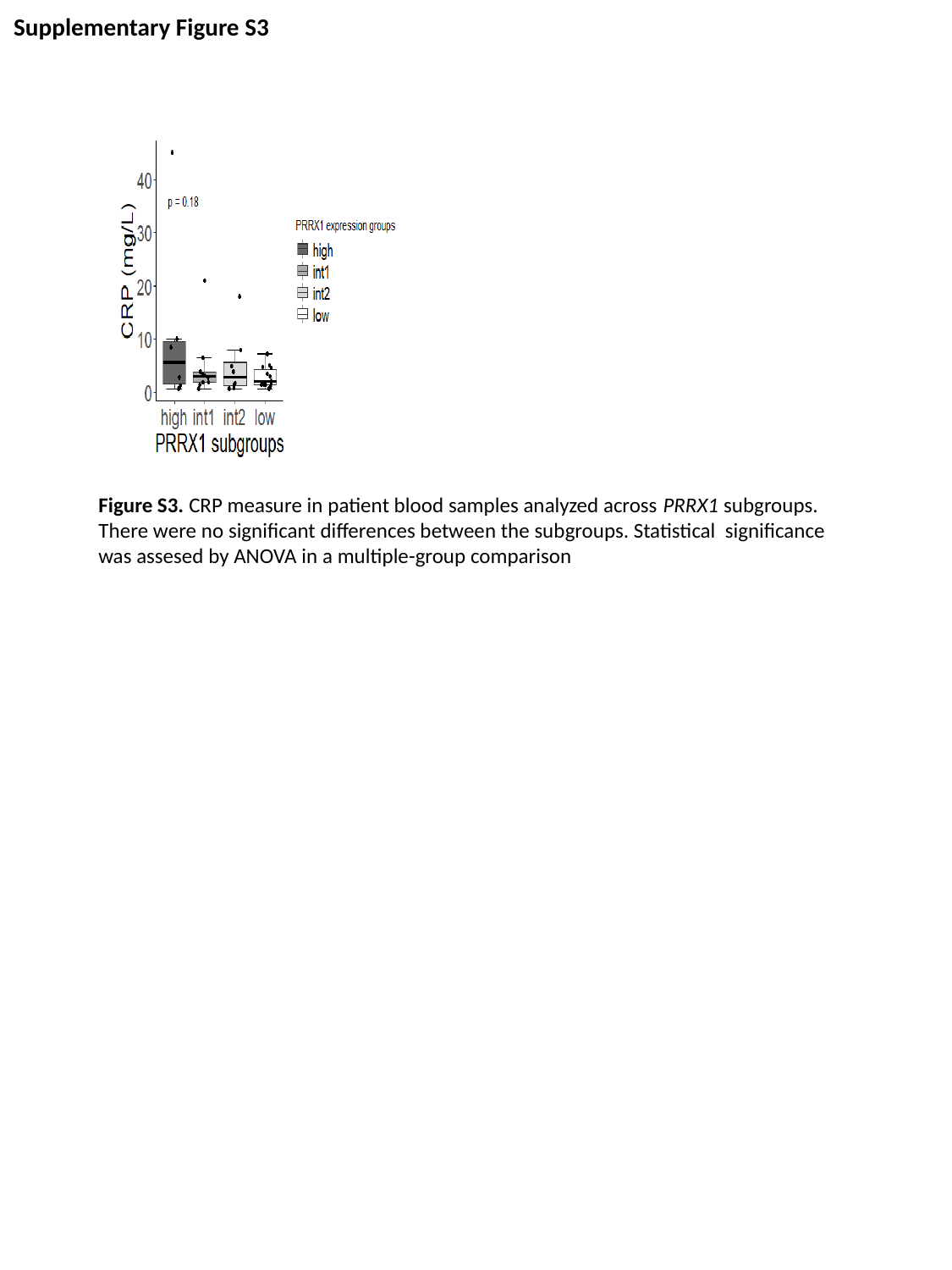

Supplementary Figure S3
Figure S3. CRP measure in patient blood samples analyzed across PRRX1 subgroups. There were no significant differences between the subgroups. Statistical significance was assesed by ANOVA in a multiple-group comparison
